# Cloud edge enabled stacked ensemble learning framework with meta model for situation aware maritime traffic monitoring and control systems

**DOI:** 10.1038/s41598-025-25020-5

**Published:** 2025-11-20

**Authors:** Zulfiqar Ahmad, Jung Taek Seo, Seungho Jeon

**Affiliations:** 1 Department of Computer Science and Information Technology, Hazara University, Mansehra, 21300 Pakistan; 2 Department of Smart Security, Gachon University, 1342, Seongnam-Daero, Seongnam-Si, Gyeonggi-do, Republic of Korea

**Keywords:** Deep learning, Maritime, Traffic monitoring, Control systems, Ensemble learning, Cloud computing, Edge computing, Computational science, Computer science, Information technology

## Abstract

In the last few years, the increasing trend of vessel density, different types of vessels, and the increased need for real-time data have made maritime traffic management significantly more difficult. This study presents a situation-aware framework based on stacked ensemble learning and cloud-edge hybridization, which is aimed at enhancing the maritime traffic monitoring and control systems. This approach combines stacked ensemble learning with a meta-model for vessel type classification and employs the concept of cloud-edge architecture to strike a balance between computational efficiency and delay minimization. While the edge layer takes care of real-time inference and situational analysis on the go, the cloud layer takes care of model training and amalgamation of data from various sources. Our evaluation made use of a comprehensive maritime vessel dataset and compared the performance with the state-of-the-art deep learning models (VGG16, VGG19, DenseNet121, and ResNet50). Our experiments show that the stacked ensemble learning with a meta-model significantly outperforms the traditional ones, achieving an overall accuracy of 0.98, macro average precision of 0.97, macro average recall of 0.98, and an F1-score of 0.98. Both ROC and PR curves also demonstrate excellent AUC values, which tend to 1.00 for almost all categories of vessels, which is a strong performance in distinguishing vessels from each other. Test predictions are outstandingly accurate, with confidence in vessel classification exceeding 99% in most cases. From these results, the proposed method shows robustness, scalability, and effectiveness for real-time maritime surveillance, naval defense systems, and autonomous vessel traffic control in industrial IoT environments.

## Introduction

Maritime environments refer to the vast and constantly changing waters that encircle the world; they are a vital component of global commerce, travel, and many biological processes^1,2^. The maritime environments are influenced by their flowing water, fluctuating tides, complex meteorological conditions, and the great variety of operations under and above the water. The global economy is heavily reliant on the maritime sector, which helps to move goods, fuel, and resources around the world. Maritime environments support numerous activities, including shipping, fishing, military operations, and tourism, each of which is defined by its unique environmental setting at sea^3,4^. Effective stewardship and awareness of marine environments are prerequisites for the safety, productivity, and sustainability of the industries concerned and for supporting the health of aquatic ecosystems^5–8^.

Besides their ecological advantages, maritime environments are significant pillars of the global economic system, as they shape commerce and energy supplies and telecommunications. Sea transport carries over 90% of global commerce^9^, and maritime paths are indispensable to make the expansion of the economy and creation of wealth possible. The seas and oceans are supporting significant scientific initiatives, facilitating the monitoring of climate change, and providing natural resources like fisheries and minerals. As vital to economics, as strategically important, and as ecologically healthy, maritime environments are worth protecting for sustainable development and stability of the globe^9,10^.

Maritime traffic monitoring and control systems (MTMCS) support the safe and sustainable passing of vessels around the world. The tracking of the positions, speeds, and trajectories of vessels is performed using radar, satellite surveillance, AIS, and marine climate data that are incorporated in these systems^11,12^. Integrating information from various platforms, MTMCS contributes to real-time maritime situational awareness, improved navigation decisions, and the prevention of vessel collisions, groundings, and accidents^13–15^. In heavily congested waters around ports, shipping channels, and straits, these platforms are very useful because of the increased probability of collisions due to high vessel density. These systems help achieve environmental sustainability by monitoring pollution potentials in water, fuel consumption, and emissions. The combination of cloud-edge hybridization and ensemble learning in these systems is resulting in greater adaptability, efficiency, and better capacity in predicting traffic, vessel routing, and ensuring safety and security at sea^16–19^.

Deep learning architectures, such as VGG16^20–23^, VGG19^24–26^, DenseNet121^27–30^, and ResNet50^25,31–33^, have great abilities to improve the maritime vessel detection and classification field, which has led to a high increase in terms of precision and efficiency^34–36^. Because of their ability to analyze complex data from images and sensors, these networks are extremely appropriate for maritime environments. Admired for classifying accuracy and skill in pattern identification, VGG16 and VGG19 operate as deep convolutional neural networks capable of differentiating objects from images at multi-scale resolutions. They facilitate the exact identification and classification of ships, such as cargo ships, tankers, and fishing boats, from satellite or aerial sensor-collected data. Due to their layered structure, they can extract fine-grained spatial representations from vessel imagery, tolerate diverse illumination, and facilitate reliable mechanical vessel monitoring in dynamic maritime environments. Another creative CNN architecture is DenseNet121, which gets its uniqueness via a mechanism that connects each layer to all others in the network. DenseNet121 excels in extracting meaningful information from complex maritime environments because of its dense architecture that fosters efficient feature sharing. This architecture is highly effective in vessel detection in adverse conditions such as fog, strong winds, or bad lighting. The robustness of the model to environmental changes makes it indispensable to the maritime traffic monitoring systems, which frequently encounter dynamic conditions^37–40^. The thorough analysis of the feature offered by DenseNet121 allows for the separation of the closely related vessel categories and, as a result, increases the accuracy of vessel classification. With the help of residual connections, ResNet50 reduces the risk of vanishing gradients in training, enabling the network to capture richer data features, which surpasses common limitations of deep neural architecture. The effectiveness of ResNet50 in maritime applications is significant, particularly when the vessel density is high and when vessels are visible in individual frames together. The additional depth of the network gives it the power to recognize small differences between the outlines, sizes, and motion patterns of vessels irrespective of how complex the visuality is, which increases the trustworthiness and accuracy of vessel classification systems^16,41–43^.

Stacked ensemble learning with a meta-model is exceptionally beneficial to maritime vessel detection and classification, as models can learn to instantly adapt to new conditions without having to be retrained almost from scratch^44,45^. Many of these already existing deep learning methods are not suitable for managing diverse data sets or adapting to changes in environmental settings. Ensemble learning application enables models to quickly re-learn based on the experiences of prior events. Such a task ability is particularly helpful in the detection of maritime vessels since maritime environments are always affected by changing weather, water conditions, and the presence of various types of vessels. To be specific, a vessel detection model developed in ideal weather conditions may not perform equally well in adverse or unpredictable weather conditions such as fog and storms. By ensemble learning, the model can adjust its detection procedures to previous exposures and, as such, become better at dealing with new environmental changes. The most important advantage of ensemble learning is that it increases the efficiency of the detection system. Maritime data collections are voluminous and complicated, with multiple sensing techniques such as radar and optics and a variety of environmental conditions^46^. Ensemble learning application assists the model to generalize better, which is a reduced need to retrain or to obtain a lot of labeled data. This is very important because of the scarcity or high cost of acquiring annotated data in maritime vessel detection. This technique is the foundation of few-shot learning in ensemble learning, i.e., the model can be trained to recognize new vessel types or atypical situations with a small set of labeled instances. Specifically, this benefit is of great importance for processing newly introduced vessels in the maritime industry or when dealing with the uncommon maritime traffic scenarios that are not represented in training sets^47,48^. The use of deep ensemble learning leads to more robust maritime vessel detection systems. Maritime environments are usually difficult due to weather interference, reflections from water, or an overcrowding of ships. Because of exposure to a broader range of scenarios, ensemble learning helps models maintain their accuracy of detection in noisy and unpredictable environments. The system is able to change its approach based on feedback collected from previous detection exercises and hence maintain its performance in dynamic maritime environments. This sort of change boosts precision detection and enhances the general reliability of maritime monitoring systems, able to cope with the regular and diverse issues of maritime operations^10,49,50^.

Deployment of cloud-edge computing in maritime traffic monitoring and control systems is an important first step towards increasing operational efficiency, scalability, and responsiveness in maritime operations^51,52^. Centralized storage, high-performance computing, and continuous operation typical of cloud computing allow for extensive data analysis, real-time decision-making, and use of high-level modeling techniques over the wide expanse of the maritime environment^53,54^. However, since maritime environments are vast and not well developed, near-source real-time processing is often a necessity. Edge computing is, in fact, particularly helpful here: With edge computing, doing calculations on the data source or near the source (on ships, nearshore infrastructure) reduces latency and safeguards network resources and strengthens immediate response capabilities. While the cloud focuses on secondary issues, edge computing deals with pressing operations in the maritime traffic systems, including vessel surveillance, flagging unusual behavior, and neutralization of local threats^55–57^. Through the synergy of cloud and edge computing, maritime traffic systems may realize scalability and responsiveness and hence provide seamless vessel coordination and governance in diverse and large maritime regions.

The motivation behind this research lies underneath in the increasing complexity and demands experienced by current maritime traffic management and control systems. With the increased global maritime traffic, the systems should be efficient, able to provide real-time performance, and scalable in order to support safety, security, and environmental sustainability. Latency, the availability of computational resources, and network bandwidth are traditionally problematic for maritime monitoring systems, becoming particularly significant in remote or large-scale maritime regions. A feasible solution to these concerns can be achieved by integrating cloud-edge computing with novel deep learning techniques. Using the synergy of cloud and edge technologies, the research aims at developing a system that can process expansive and varied maritime traffic data, process it in real-time, and provide immediate and accurate guidance for the managers of maritime traffic. Using deep neural networks for vessel detection and classification and ensemble learning for adaptability significantly increases the readiness of the system to handle unexpected and dynamic maritime circumstances. Our research aims at enhancing maritime traffic management with more reliable, efficient, and intelligent solutions to minimize risks and enhance the efficiency of navigation amid increasing marine traffic complexities and variance. The main contributions of this article are highlighted below:

- We proposed a novel framework that combines deep learning models to make stacked ensemble learning with a meta-model and provide real-time situation-aware maritime traffic monitoring and control systems. Our framework is designed to accommodate various maritime scenarios, such as differences in the type of vessels, environmental conditions, and patterns of traffic to ensure strong performance and efficiency during live operations.
- We have implemented advanced deep learning architectures, such as VGG16, VGG19, DenseNet121, and ResNet50, for the enhancement of vessel detection and classification accuracy and the level of reliability. With these models, we examine maritime inputs, identify key aspects, and precisely differentiate between different vessel categories.
- Our work introduces stacked ensemble learning with a meta-model that increases the system’s ability to adapt to unfamiliar maritime environments. Its capacity to leverage ensemble learning makes our framework efficient with emerging environmental factors like varied weather, vessel characteristics, and unexpected traffic conditions, with minimal retraining.
- We provide the practical mechanism of incorporating cloud-edge hybridization into maritime traffic management. By utilizing cloud and edge technologies simultaneously, maritime traffic systems can process and analyze information quickly with the best computational support. Non-critical data storage and analytics are delegated to the cloud for scalability, but edge computing nodes facilitate real-time action and quick response to maritime crises such as vessel accidents or changes in traffic.

The rest of the paper is organized as follows: SectionRelated work provides the related work. In SectionSystem design and model, system design and model are described. In Section Algorithm for the proposed framework, the performance evaluation is highlighted, and in the last section, Section Performance evaluation, we conclude the article with some future directions.

## Related work

We reviewed the related work in respect of traffic monitoring and control systems in maritime environments, implementation of deep learning architectures and cloud-edge hybridization.

Details of a video-based ship tracking approach that is to enhance the maritime traffic situation intelligence under harsh weather conditions are provided in^58^. A combination of multi-view learning and particle filtering is used by the framework to accurately estimate the positions of ships in surveillance videos. In order to increase the reliability of tracking, an outlier suppression technique based on a Kalman filter was introduced into the system. Validation results under scenarios of ship occlusion, sea clutter, and small vessels have provided evidence of method effectiveness in enhancing reliability of visual tracking for maritime surveillance. In^59^, the study is proposing a robust data fusion scheme to enhance maritime traffic monitoring capabilities in inland waterways. The approach involves integrating Automatic Identification System data and visual footage from cameras placed along the shoreline. AIS input provides vessel identity and motion information, and visual data improves appearance-based visual tracking. To overcome challenges like occlusion and data inconsistencies, the authors proposed an anti-occlusion tracking method that included the use of DeepSORT and a Hungarian algorithm that combined several features. A coordinate transformation technique was applied to align AIS geographic data with the camera-imaged plane. By combining AIS and visual data, the proposed approach significantly improved the precision of tracking and the identification of vessels, confirmed through the use of the data from a new multi-sensor dataset, and supported better maritime traffic safety and efficiency.

The study in^16^ responded to the immediate need for exact ship speed prediction for effective maritime operations planning and traffic control in the Saint Lawrence Seaway. Rather than taking fixed assumptions of vessel speeds, the research used historical voyage records and used deep learning models that were tailored to each type of vessel and route. In comparison of three sequence learning frameworks, the presented method displayed higher predictive accuracy than the current standard methods. The adoption of deep learning in this study presents promising results to deliver the current ship speed forecasts route-by-route, which can promote operational efficacy, safety standards, and environmental assessment at sea. In their work^60^, the researchers proposed an advanced trajectory prediction framework for maritime IoT that can address the problems associated with explosive growth of vessel trajectory data collected by AIS base stations using satellite technology. The exponential growth of data pointed to major hazards to maritime safety and efficiency in operations. In order to improve smart traffic services in maritime IoT, the authors used deep neural networks, primarily Long Short-Term Memory (LSTM) networks, to provide accurate spatiotemporal predictions. Vessel traffic conflict modeling developed based on dynamic AIS data and social force theory is a critical factor in enhancing the predictive accuracy of the model. In order to obtain uniform performance in various maritime settings, a mixed loss function was introduced. Its effectiveness was confirmed by thorough testing with actual AIS data showing the possibility of enhancing vessel route prediction for avoiding collisions, monitoring, and detecting unusual vessel activities.

The proposed method in^61^ involves a bifurcated vehicle control system that is based on both edge and cloud computing for better remote control in a maritime environment. The system is able to compensate for the challenges arising from climate-related and water dynamics as well as the constraints on available wireless connections. For the non-ideal, model-free situations, the research instituted Deep Reinforcement Learning (DRL) with the use of the Soft Actor-Critic (SAC) algorithm. Resident models at the edge server provide rapid control decision-making, while the cloud server uses lifelong learning to iteratively optimize the resident model accuracy. To improve lifelong learning on the cloud server, the authors developed the version of model (VoM) concept and weighted sampling scheme. Moreover, the authors presented a dual window updating strategy meant to improve the streamlining of learning through the use of a sliding and freeze window technique that minimizes redundancy of adjustments. Simulation in a pendulum model and a remotely operated vehicle (ROV) validated the method’s capacity to adapt to maritime environmental conditions. The latest progress in machine learning and deep learning has had a great impact on the creation of intelligent systems in the maritime environment, especially on operations that required the classification of seabed sediments, the prediction of sensor performance, and acoustic image analysis. The application of ensemble learning to solve the problems related to complexity and noise in sonar and marine data has been investigated by several works. As an example, in^62^, a stacking ensemble learning approach was used to classify seabed sediment based on sonar imagery. A meta-learner was constructed to combine predictions of base models, which gave higher accuracy and robustness of classification than conventional models. This proves the possibility of ensemble architecture when dealing with the variability of acoustic marine data. In addition to sediment classification, the ensemble methods have been utilized in the Internet of Everything (IoE) setting in predictive maintenance and optimization of resources.

In^63^, the framework of deep ensemble based on the meta-learning concept was proposed to predict the remaining battery life (RBL) of IoE sensors. The suggested MetaStackD model used several regression algorithms (Random Forest, Gradient Boosting, and LightGBM) to approximate the intricate correlation between sensor workload and power consumption. The method obtained a remarkable accuracy and computational efficiency that underscores the suitability of the ensemble learning in energy-restricted and changing maritime sensor networks. The study in reference^64^ introduced a powerful deep learning framework ensemble that was introduced to enhance the confidence of acoustic seabed sediment classification. The framework solved some of the general problems that are related to data variability, misclassification, and generalization error by integrating multiple deep models. The findings demonstrated that the ensemble deep learning model performed better than individual models and classical ensemble methods in accuracy and stability. In^65^, the authors proposed a Bidirectional Motion Temporal (BiMT) approach for automated solutions to improve accuracy and efficiency by coupling a pre-trained model of deep neural networks. The system uses CNN to extract the best spatial key features from the video stream, which are transformed into time series data and coupled with Recurrent Neural Network (RNN) deep learning model. Bidirectional Long Short-Term Memory (BiLSTM) extract temporal key features, and Multi-Head Self-Attention (MHSA) permits for the identification of short-term frame correlations. Categorical focal loss (CFL) was used during training to prioritize essential features and to further enhance the precision.

Recent advancements in the research of maritime traffic monitoring and control have made remarkable progress in the use of deep learning, data fusion, and multiple computational models to improve vessel tracking, predictability, and decision support. Approaches to maritime traffic monitoring and control have included video-based tracking, data fusion for vessel recognition, model construction for speed prediction, and machine learning for trajectory forecasting. Though these methods provide important findings, they have inherent limitations that limit their practical use in real-time, variable maritime conditions. Much of the research is focused on specific aspects of traffic management, such as ship tracking or speed forecasting, but hardly ever works with the integrated needs for timely traffic control and informed decisions. Most of the existing systems emphasize narrow scopes of maritime traffic management, which means that they fail to provide a sufficient basis for context-aware decision-making in response to such variables as vessel type, real-time weather, and traffic conditions. Most of the existing systems mostly use cloud or edge computing instead of a hybrid combination of their respective advantages. Maritime environments, which are typically found far away, present latency and bandwidth challenges to cloud-based systems, and edge-based systems may not provide the level of scalability and computing capability that is required for the large amount of maritime data processing. The existing inadequacies in hybrid cloud-edge systems for real-time maritime traffic management prevent the practical scalability and timely response abilities of existing systems. Our research aim is to address these gaps by introducing a revolutionary stacked ensemble learning-based situation-aware framework based on cloud-edge hybridization technology. By employing cutting-edge deep learning architectures, meta-learning principles, and cloud-edge infrastructure, our framework enables a responsive, flexible system customized for real-time maritime traffic management. The proposed framework makes use of vessel classification, context-aware decision-making, and efficient data processing in hybrid cloud-edge architectures in order to enable timely and accurate decisions even in harsh and isolated maritime environments. The outcome system significantly improves maritime traffic management by improving safety, operational efficiency, and responsiveness to changing situations.

## System design and model

**Fig. 1:**
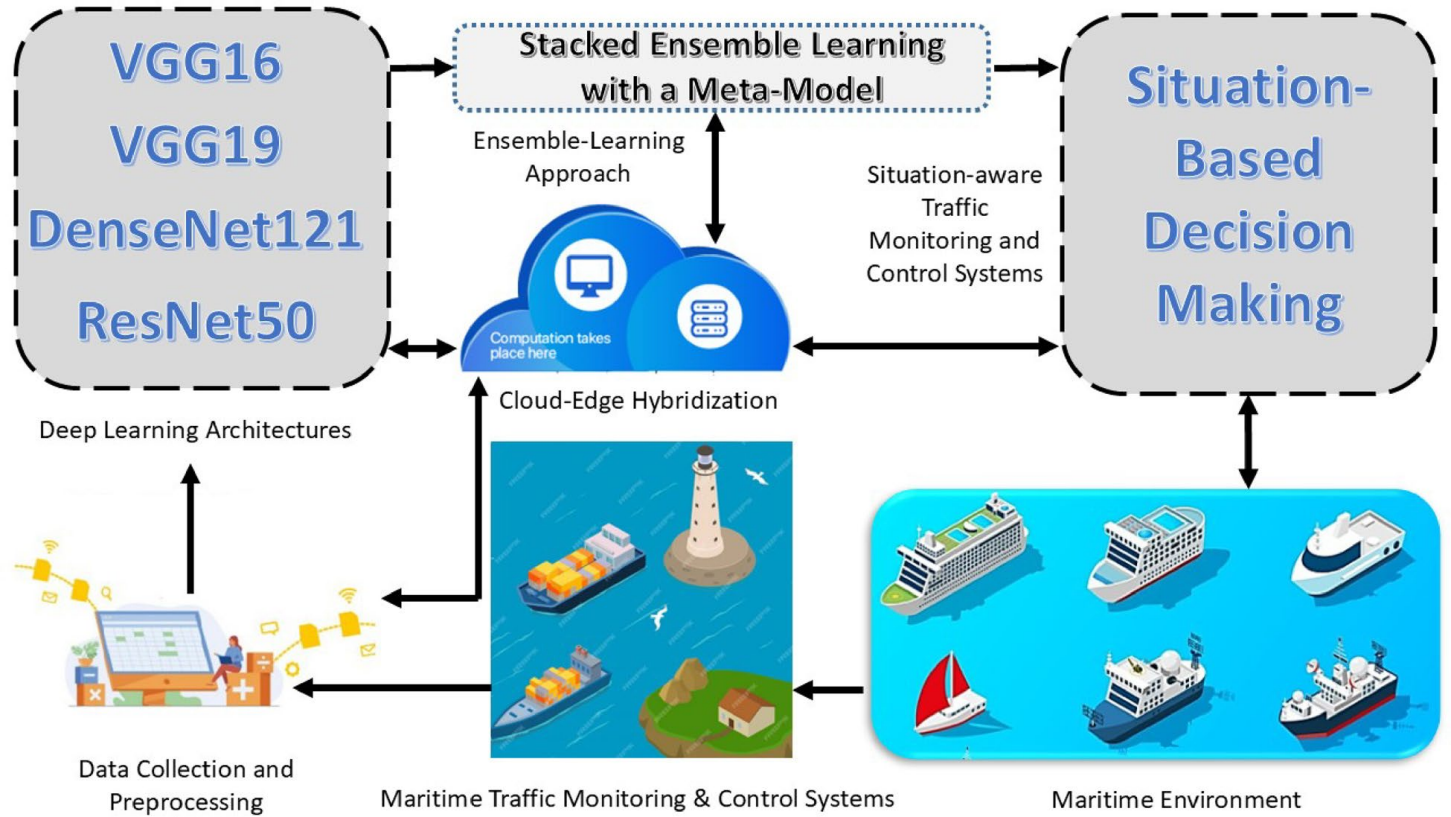
Maritime traffic monitoring and control system using stacked ensemble learning with a meta-model.

In this research, we present a Cloud-Edge Enabled Stacked Ensemble Learning Framework with a Meta-Model for Situation-Aware Maritime Traffic Monitoring and Control Systems. The framework seeks to provide real-time monitoring and classification of maritime vessels for decision-making to enable efficient and intelligent control of maritime traffic. Through the integration of advanced deep learning methods, stacked ensemble learning with a meta-model, and cloud-edge integration, the framework provides trustworthy, customizable, and extendable applications for the management of dynamic maritime environments. As can be seen in Fig. 1, the proposed framework consists of several integrated components. The framework starts with the maritime environment, which includes data input of images. Through constant gathering and analysis of this data, one maintains a full picture of the maritime situation. Data Collection and Preprocessing: In the data collection and preprocessing stage, the collected data is cleansed and normalized, and features of meaningful data are extracted. It is at this point that the data is made fit for use in deep learning models by cleaning it of noise and maximizing its quality. The Deep Learning Architectures Implementation phase takes in and processes the preprocessed data. Four state-of-the-art deep learning architectures are applied here, including VGG16, VGG19, DenseNet121, and ResNet50, for recognizing and identifying multiple vessel categories based on the analyzed data. Using the analysis of images, radar signals, and feedback from sensors, these models make it possible to identify types of vessels, their locations, and other important features. By obtaining important features from the data, these models ensure proper and efficient classification of vessels under challenging maritime contexts.

We utilize multiple pretrained models (DenseNet121, ResNet50, VGG16, and VGG19) to extract features. The extracted features are concatenated and passed to a meta-model (dense neural network) for final prediction. This approach follows the stacking technique in ensemble learning, where the base models act as feature extractors and the meta-learner combines these features for improved classification. This mechanism enables the system to respond quickly to unknown situations, drawing from what it learned from past records. Powered by a meta-model, the system acquires the capability to iteratively improve the route to decision-making and is thus prepared to cope with maritime situations with varying classes of vessels, chaotic weather, and changing traffic situations. The situation-based decision-making components use the vessel type and location-specific data to make decisions about route optimization, collision avoidance, and overall vessel control. The decision-making system suggests the optimal way of controlling traffic congestion, reducing environmental issues, and minimizing other risks, thus supporting safe and efficient maritime operations. All elements of the framework (data collection and preprocessing, deep learning implementation, meta-model implementation, and decision-making components) correspond to the cloud-edge hybridization model. Cloud computing facilitates extensive data handling and distance storage, and edge computing nodes guarantee fast, low latency processing close to the originating source of the data. This model is able to handle large volumes of data in a timely manner and ensure that important decisions are made in a timely manner, thus minimizing response time in real-time situations. The steps for cloud-edge hybridization mechanism are described below:

- The framework enables localized analysis at edge nodes such as onboard processing units, fog nodes, or maritime gateways. This approach minimizes latency and enables immediate situational responses, especially in scenarios such as vessel collisions, route deviations, or rapid traffic changes.
- Edge devices are utilized to perform early-stage data preprocessing tasks including cleansing, feature extraction, normalization, and dimensionality reduction. This minimizes network overhead and ensures only relevant, refined data is transmitted to the cloud, leading to optimized bandwidth utilization and reduced congestion.
- The cloud environment is reserved for high-computation tasks such as training and inference of deep learning models, ensemble learning integration, and long-term storage. It ensures scalable processing for predictive analytics, historical trend analysis, and centralized decision support.
- Based on hybrid inputs from both edge and cloud layers, the framework provides real-time feedback and decision outputs (e.g., vessel classification labels, control recommendations) for proactive maritime traffic management.

We chose a stacked ensemble learning–based meta-model for ship classification to effectively integrate the predictions from multiple diverse CNN models. Each base model captures different feature representations, whereas the stacked ensemble with a meta-model learns to optimally weight their contributions for the final prediction. Unlike bagging, which reduces variance by applying the same model to different data subsets, or boosting, which sequentially corrects errors, the meta-model directly learns the relationships among the outputs of the base models. This approach provides higher accuracy, robustness, and flexibility for complex multi-class classification tasks. The framework allows for efficient operation in remote marine areas, compensating for network latency and bandwidth issues, typical with standard cloud solutions. The development of a complex and flexible solution for maritime traffic monitoring and control has been achieved by using a blend of advanced deep learning, ensemble learning, and cloud-edge hybridization. Using advanced real-time analysis, advanced vessel identification, and context-driven decision-making, the system progresses maritime safety, simplifies operations, and reduces response times in unpredictable situations.

## Algorithm for the proposed framework

Algorithm 1 is based on the ensemble deep learning approach with cloud-edge hybridization, setting out a full solution for maritime traffic monitoring and situational control. Stakeholders start by gathering and preparing real-time maritime information by obtaining information from radar, satellite resources, and onboard sensors and then forwarding it to the nearest edge computing center for further analysis. At each edge node the data is cleaned of noise, normalized, and resized to the proper dimensions to preserve the data quality before deeper processing. At the feature extraction and vessel detection stage, four state-of-the-art deep learning models, which include VGG16, VGG19, DenseNet121, and ResNet50, are implemented to analyze the preprocessed data. After each model generates a deep feature representation in isolation, these are combined using ensemble or fusion style methods to produce a rich, unified feature vector. This resulting unified feature vector is then used as an input to a SoftMax-based output layer that classifies maritime vessels into their type and operational profile.

To enhance the capacity of the system to react to changing maritime conditions, an ensemble learning module has been introduced. This component is based on a spectrum of analogous tasks for the rapid tuning of model parameters, which quickly adapts to unknown or shifting maritime conditions. When adapting ensemble-parameters over a variety of learning tasks, the system is robust, enabling rapid retraining to new or previously unrecognized vessel categories with minimal supplementary training data. Once vessel types have been established, the framework transitions to context-dependent decision-making. While keeping in mind real-time context such as weather, position, and traffic volume combined with vessel type, the system rolls out rule-based or learned decision strategies to determine the best control options. The system can suggest providing warnings, suggesting the vessels change their speed, showing them new routes, or introducing systems to avoid collisions. The system uses cloud-edge hybridization to enhance both computational speed and real-time response times. The cloud performs bulk training, ensemble learning progression, and consolidation of data streams, while edge devices handle critical operations such as initial data cleaning, real-time analysis, and speedy control instructions. Constant synchronization of deep models and decision modules from the cloud to the edge keeps the system prepared and flexible, which allows the system to efficiently handle future developments. Such an approach provides for the deployment of a robust, smart maritime monitoring system that responds quickly, classifies vessels correctly, and makes informed control decisions.

**Algorithm 1:**
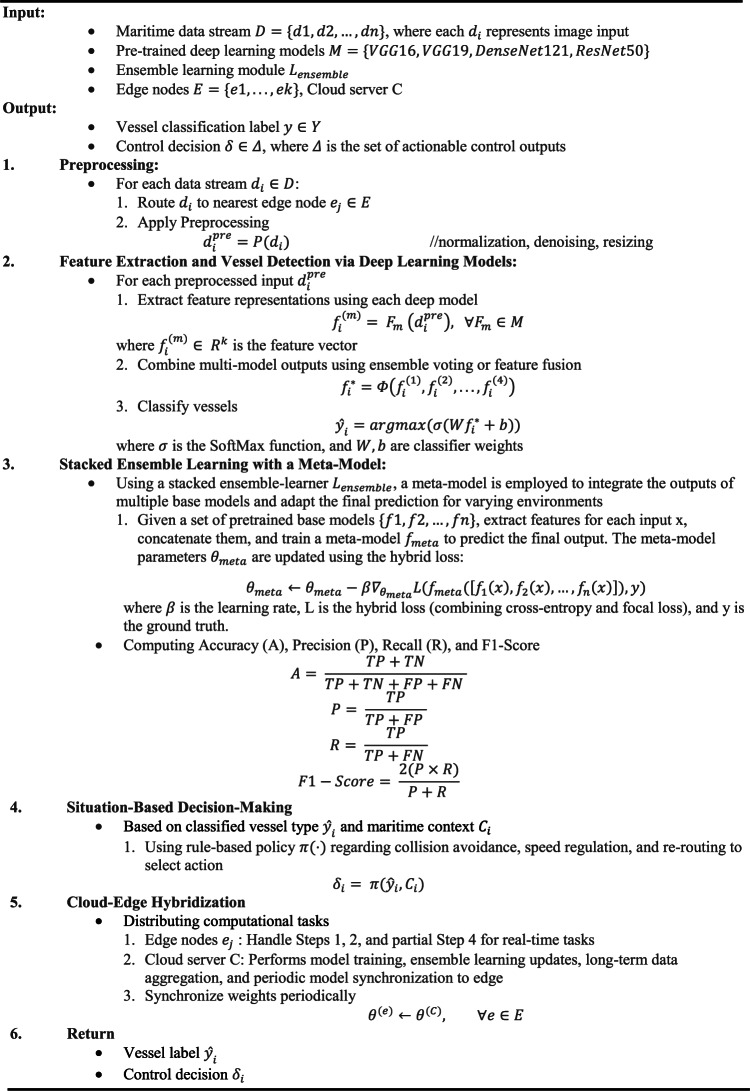
Maritime traffic monitoring and control using stacked ensemblelearning with a meta-model and cloud-edge hybridization

## Performance evaluation

We perform simulations and evaluate the performance of the proposed framework as given below:

## Evaluation metrics

We evaluated the performance of the models implemented in the proposed framework using precision, recall, F1 score, accuracy, ROC curve, and PR curve. We calculated accuracy, precision, recall, and F1 score based on the terms True Positive (TP), True Negative (TN), False Positive (FP), and False Negative (FN). TP is the number of correctly identified positive instances. TN is the number of correctly identified negative instances. FP is the number of incorrectly identified positive instances. FN is the number of incorrectly identified negative instances.

## Dataset

We have used the Ship Classification Dataset from Kaggle^66^ for our work. The dataset has been selected for the purpose of detection and classification of maritime vessels. This collection contains more than 4000 annotated images that represent a wide range of ships, cargo vessels, tankers, warships, sailboats, and cruise liners. The dataset has significant value for the development and evaluation of deep learning architectures in classification applications because of the organization of the images by visual attributes and functional classification. Diversity of backgrounds, lighting scenarios, angles of view, and sizes of vessels in the set improve the generalization capability of the machine learning and deep learning models in real maritime environments. We selected this dataset because it was relevant to our objectives due to the diversity of its content and the accuracy of the labels. Due to the vast range of vessel classes covered in the dataset, we are able to compare and apply various deep learning networks, including VGG16, VGG19, DenseNet121, and ResNet50, which enables a thorough performance evaluation. The acquisition of real-world maritime images also promotes the development and training of ensemble learning framework that works well in various maritime situations. The fact that its open dataset is available, the high-quality annotations it has, and the availability of a large dataset make it the preferred choice for developing and evaluating our proposed situation-aware, cloud-edge hybridized framework.

## Experimental design

The experimental evaluation was conducted by implementing four deep learning architectures, VGG16, VGG19, ResNet50, and DenseNet121, integrated within ensemble learning-based framework. The dataset was partitioned into three subsets: 80% for training, and the remaining 20% equally split into 10% for validation and 10% for testing. All experiments were executed in a GPU-enabled Python environment, leveraging widely used machine learning and data processing libraries such as TensorFlow, Keras, Pandas, NumPy, Seaborn, Scikit-learn, and Matplotlib. Preprocessing techniques including Label Encoding and One-Hot Encoding were applied to prepare the data for model training.

## Data preprocessing

**Fig. 2:**
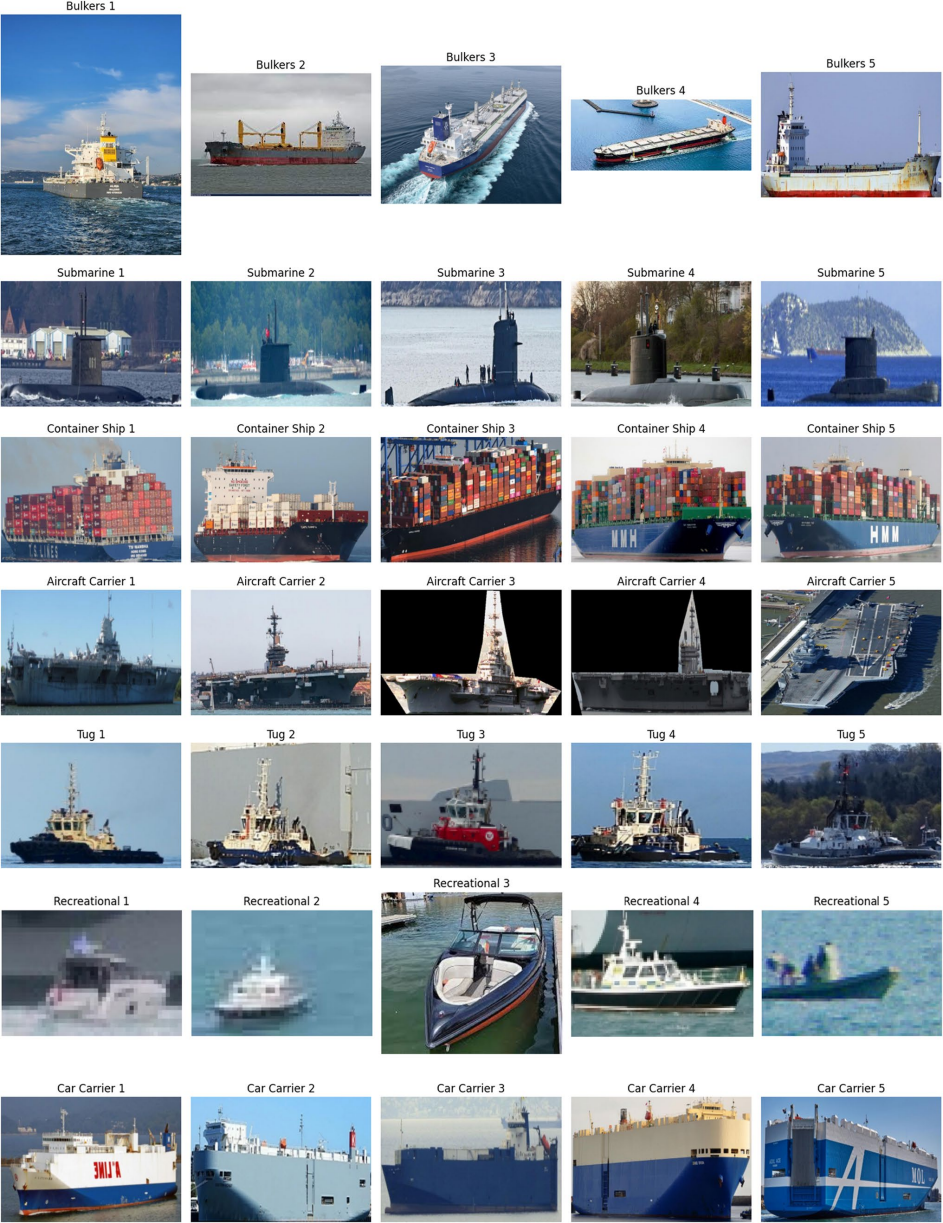

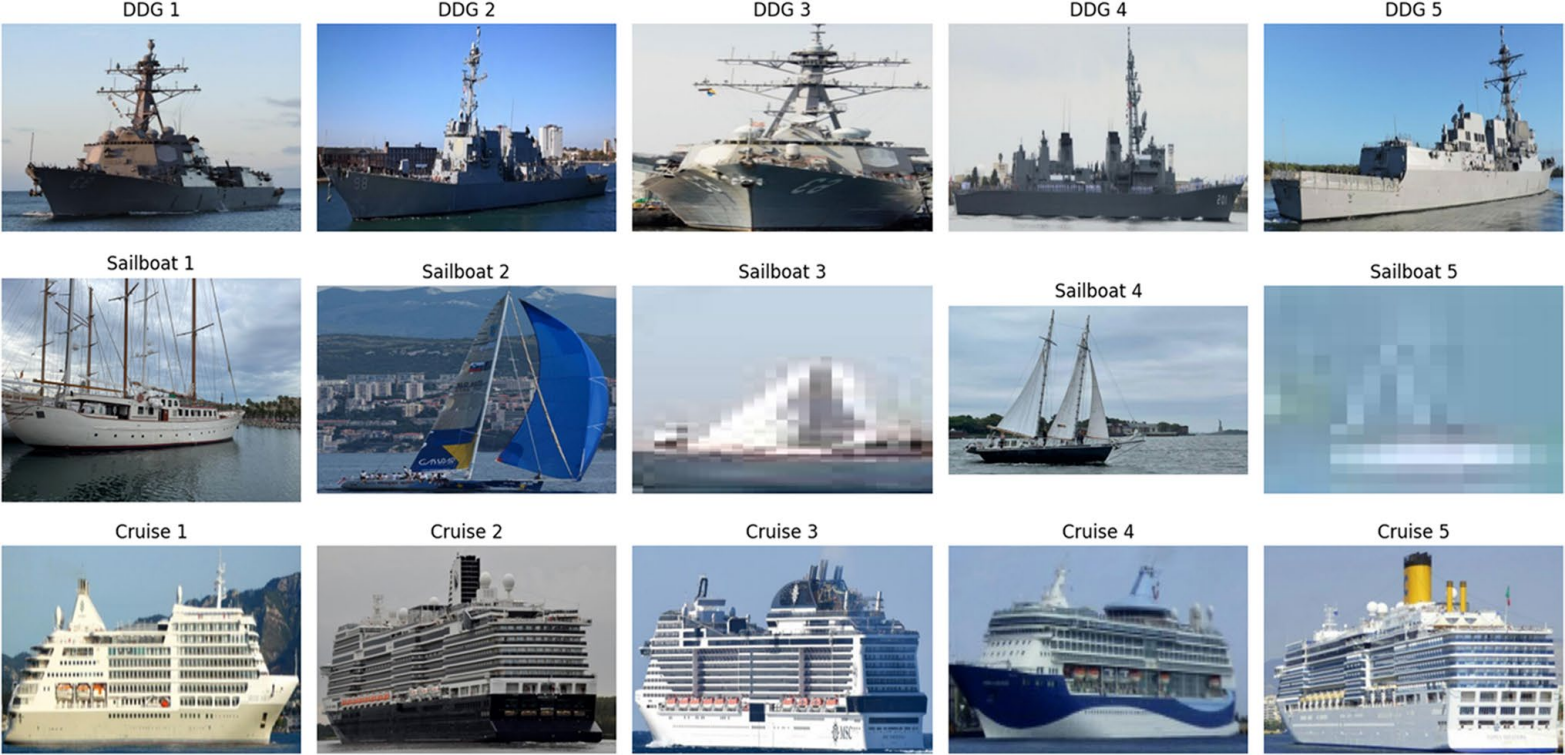
Sample images for each category.

We performed a thorough review of the dataset used for ship classification. The data collection was arranged such that the images would be labeled and sorted into folders for each category of ship type: aircraft carrier, container ship, cruise, DDG, recreational, sailboat, submarine, tug, car carrier, and bulker. To check whether representative samples were found within each class, a custom function was created for dataset structure inspection with results shown in Fig. 2. To maintain transparency and clarity, images at the start of each category were selected and visually examined. Subsequently, all image file paths and relative labels were collected and arranged in a formatted pandas DataFrame. The resulting DataFrame was used to prepare the dataset for partitioning and more data transformation. Stratified sampling was performed to maintain the same original class ratio in each subset for training (80%), validation (10%), and testing (10%). By doing so, we ensured that the ship category distributions were balanced in subsets, which helped to avoid class imbalance and improve the model’s performance.

After splitting, ImageDataGenerator from the TensorFlow Keras API was applied to perform the required image data preprocessing. Two separate generators were defined: one generator that was set to perform augmentation only on the training set and another one that supplied validation and test images in their original form. Various real-time augmentations were used by way of the training data generator, which artificially increased the size of the dataset and helped with better generalization. These transformations included:

- Rescaling pixel values by 1/255 to normalize them to the [0,1] range,
- Random rotations up to 20 degrees,
- Width and height shift up to 20%,
- Shearing, zooming, and horizontal flipping,
- Filling strategy set to ‘nearest’ to preserve spatial integrity during augmentation.

In order to ensure consistency and prevent augmented sample data leakage, the validation and test sets were only subject to pixel rescaling. All images were normalized to the given size by img_size and colorized to RGB to be compatible with the architectures VGG16, VGG19, ResNet50, and DenseNet121. The transformation of labels to categorical form for multi-class classification was automated, and batch generation was done through the flow_from_dataframe() function while shuffling was enabled on the training and validation data. Matplotlib and Pillow were used to design a visualization pipeline for data exploration and testing the range of the dataset across ship classes. It is easy to see through visual inspection of Fig. 2 that there are five sample images for each ship class, demonstrating dataset diversity. From visual examination it was evident that the classes were distinct and that the images had differences in terms of lighting, angle, and background characteristics.

**Fig. 3:**
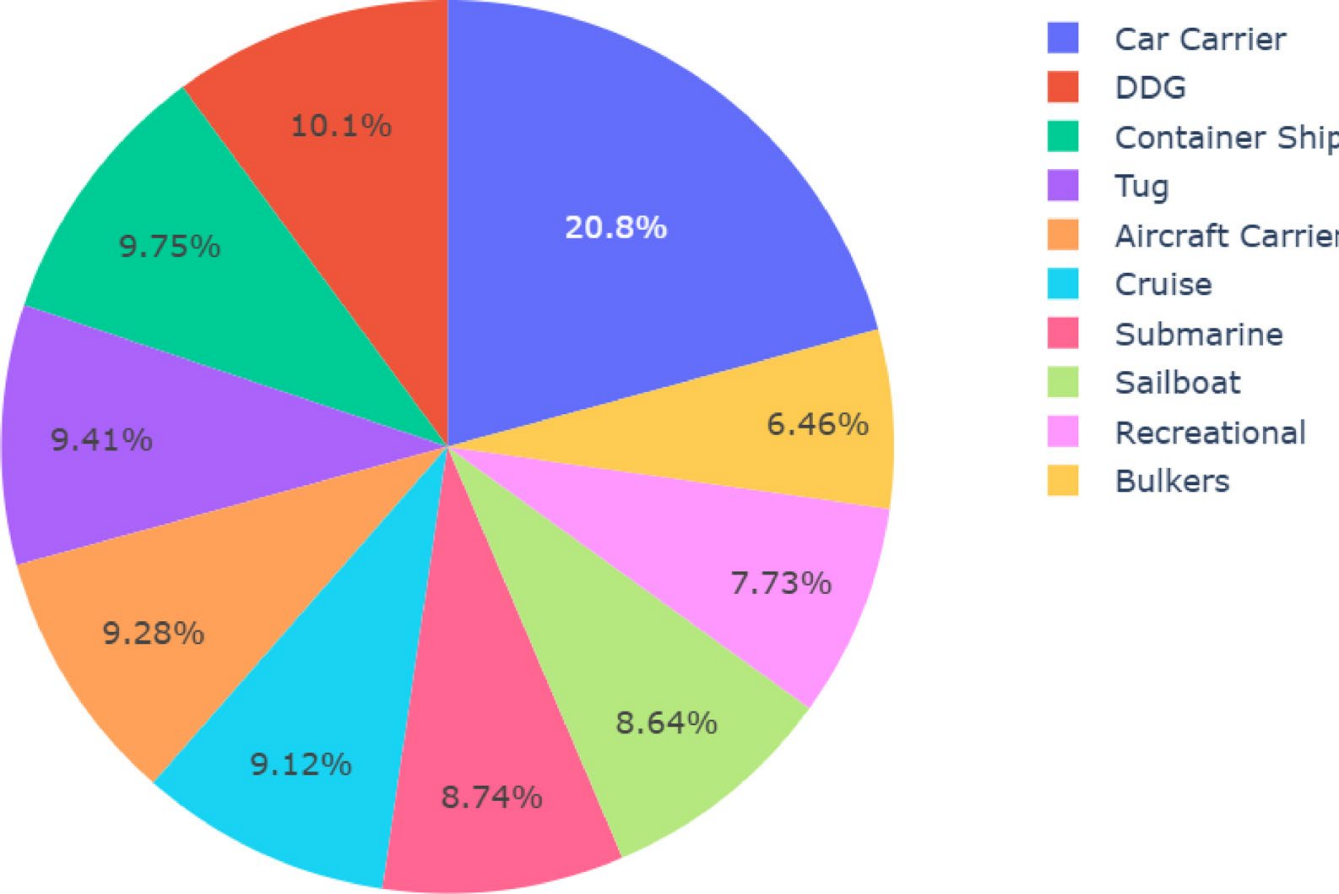
Pie chart showing the category distribution.

A pie chart indicating the distribution of labels in the dataset was developed by using plotly.express. Figure 3 showed that the dataset had a good category distribution balance, which reduced the possibility of introducing biases during training of the model. The phase used Python libraries, including Pandas for the formatting of the data, NumPy for numeric operations, Matplotlib and Seaborn for visual analysis, and Scikit-learn for dataset division and evaluation. The model architecture used the Keras API in TensorFlow and preprocessed images and used augmented data, while Pillow and OpenCV helped in loading and studying images.

Beyond the first processing stage, a conscious image augmentation strategy was applied to increase the diversity of the training set and enhance its generalization capabilities. The use of Keras’ ImageDataGenerator for in-the-loop image augmentation during training successfully discouraged overfitting so as to ensure that the model learns features across a greater range of input differences. Although class imbalance was not important, close inspection of the class distribution revealed that there was no need to apply further balancing procedures, such as oversampling. However, this magnified approach also naturally yielded a more heterogeneous data set for each class, which improved the performance and applicability to other ship types.

## Results and discussion

In this work, we used four of the most advanced deep learning models, namely VGG16, VGG19, ResNet50, and DenseNet121, and stacked ensemble learning with a meta-model, to enhance maritime traffic monitoring and classification results. These models were chosen because they have the ability to recognize complex visual patterns in images. Despite the simplicity of the design and the ability to extract hierarchical features, while VGG16 and VGG19 are important, ResNet50 is not affected by vanishing gradients as it introduces residual connections, and DenseNet121 increases feature reuse and keeps the gradient flow smooth. Considering the outstanding individual performance of the models, the ensemble learning technique was incorporated to combine their outputs to create an improved learning unit that can take advantage of their combined strengths. The ensemble learner uses the insights of the base models to make more generalizable predictions, thus increasing robustness of classification and reducing individual model bias. The construction and training of the ensemble model adhered to processes implemented in Python using Keras and Scikit-learn, where the probabilistic outputs of the underlying deep learning models were used as inputs. A performance comparison in Table 1 of a side-by-side analysis gives the performance of each individual deep learning model and the ensemble learning ensemble on precision, recall, F1-score, and accuracy, thereby validating the benefits of ensemble learning. The ensemble learning approach is distinguished by its superior and highest macro and weighted average performance on all metrics. This model recorded macro precision of 0.97, macro recall of 0.98, and macro F1-score of 0.98 with an overall accuracy of 0.98. DenseNet121 is close behind, showing competitive results characterized by a macro F1-score of 0.96 and weighted accuracy of 0.96. Despite the fact that both VGG16 and VGG19 work well, VGG16 always produces better results in most metrics compared to VGG19. In contrast, ResNet50 exhibits notable performance metric deficiencies that suggest that a little more fine-tuning or regularization might improve its results.

**Table 1:** Classification report for VGG16, VGG19, DenseNet121, ResNet50, and ensemble learning approach.

Class	Precision					Recall					F1-score					Accuracy				
	VGG16	VGG19	DenseNet121	ResNet50	Ensemble learning	VGG16	VGG19	DenseNet121	ResNet50	Ensemble learning	VGG16	VGG19	DenseNet121	ResNet50	Ensemble learning	VGG16	VGG19	DenseNet121	ResNet50	Ensemble learning
Aircraft carrier	0.99	0.97	1.00	0.83	1.00	0.91	0.81	0.92	0.68	0.97	0.88	0.94	0.99	0.75	0.99	0.96	0.92	0.96	0.84	0.98
Bulkers	0.89	0.84	0.93	0.77	0.92	0.98	0.87	1.00	0.62	1.00	0.86	0.82	0.96	0.69	0.96					
Car carrier	0.99	0.98	0.99	0.90	1.00	1.00	0.98	0.99	0.96	1.00	0.98	0.98	0.99	0.93	1.00					
Container ship	1.00	0.93	0.98	0.74	1.00	0.94	0.94	0.95	0.89	0.96	0.93	0.91	0.96	0.81	0.98					
Cruise	0.99	0.99	1.00	0.94	1.00	1.00	1.00	0.97	0.92	1.00	0.99	0.99	0.99	0.93	1.00					
DDG	0.93	0.90	0.91	0.77	0.97	0.99	0.98	0.99	0.90	1.00	0.94	0.92	0.95	0.83	0.98					
Recreational	0.87	0.79	0.90	0.87	0.89	0.92	0.91	0.91	0.83	1.00	0.85	0.87	0.90	0.85	0.94					
Sailboat	0.95	0.85	0.94	0.78	0.97	0.96	0.88	0.93	0.78	0.99	0.86	0.89	0.94	0.78	0.98					
Submarine	0.99	0.97	0.96	0.88	1.00	0.97	0.88	0.99	0.76	0.99	0.92	0.94	0.97	0.81	0.99					
Tug	0.97	0.92	0.96	0.87	1.00	0.89	0.89	0.91	0.84	0.86	0.91	0.92	0.94	0.86	0.93					
Macro avg	0.96	0.92	0.96	0.84	0.97	0.96	0.91	0.96	0.82	0.98	0.91	0.92	0.96	0.82	0.98					
Weighted avg	0.96	0.93	0.96	0.84	0.98	0.96	0.92	0.96	0.84	0.98	0.92	0.93	0.96	0.84	0.98					

When analyzed per class, the ensemble learning scores are almost perfect in a number of classes, such as aircraft carrier, car carrier, cruise, and submarine, indicating its superior generalization ability. DenseNet121 also shows excellent performance in these difficult classes. The models generate different results for classes such as Bulkers, Recreational, and Sailboat, partially due to similarities in appearance or an increased level of intra-class variance in these categories. When classifying complex classes, ensemble learning provides higher recall and F1-scores, which demonstrates the capacity of ensemble learning to operate with uncertain/difficult-to-classify images better. ResNet50 has the worst performance on these classes, suffering from a very low recall score, for instance, 0.62 for Bulkers, which leads to lower F1-scores. The evidence strongly indicates that ensemble learning is excellent at classification, especially in the context of many interrelated classes. The classifier consistently performs well in all classes, which produce minimal false positives and false negatives. DenseNet121 and VGG16 emerge as viable alternatives comparable to or greater than the ensemble learning approach. From the results, it is clear that ResNet50 needs some form of adjustment before it can adequately fit this context. This analysis justifies the advantage of ensemble learning approaches to complex tasks of visual recognition, such as identification of different classes of ships in maritime environments.

**Fig. 4:**
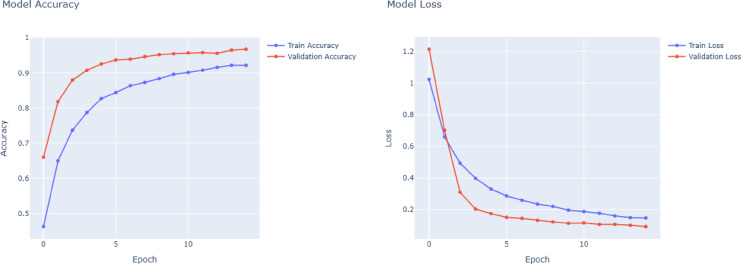
VGG16 model accuracy and model loss.

**Fig. 5:**
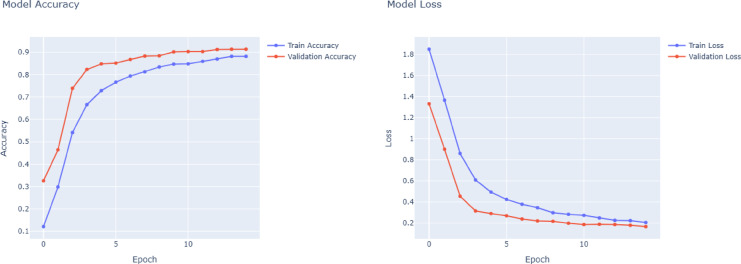
VGG19 model accuracy and model loss.

A thorough analysis of the four deep learning models, VGG16, VGG19, DenseNet121, and ResNet50, based on training curves and classification metrics reveals significant differences in performance, learning dynamics, and generalization, particularly in the context of multi-class ship classification. The training accuracy and loss curves in Figs. 4 and 7 closely match the precision, recall, and F1 score results in the results table. Among all the models, the most balanced and robust outcomes are provided by the VGG16 model. As shown in Fig. 4, VGG16’s training has a progressive and orderly increase in accuracy and systematic reduction in loss, which indicates evidence of stable convergence and effective avoidance of overfitting. This consistency is demonstrated by impressive precision, recall, and F1-scores on almost all classes, especially Car Carrier, Cruise, and Container Ship, where each class achieves close to perfection on every class. The macro and weighted averages of all performance metrics are always at 0.96, indicating that VGG16 has both balanced class performance and strong generalization in all classes. As shown in Fig. 5, the performance of VGG19 is slightly worse than that of VGG16, even though a deeper network architecture is applied. It shows macro and weighted averages (precision: 0.92, recall: 0.91, F1-score: 0.92), but classes Bulkers and Sailboat produced rather lower figures. It is probable that VGG19’s accuracy and loss curves show a lower convergence rate with a less smooth trend than VGG16, primarily due to the fact that its higher complexity may not provide consistent benefits for the size of the dataset available. One can see evidence of overfitting when the performance in the training data begins to be significantly superior to that of the validation data in the later epochs.

**Fig. 6:**
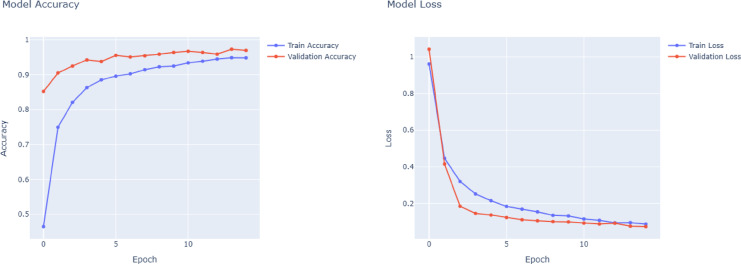
DenseNet121 model accuracy and model loss.

**Fig. 7:**
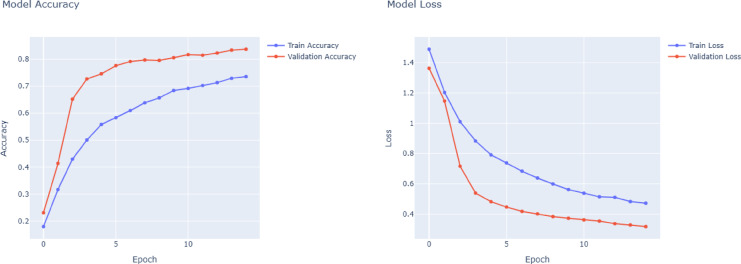
ResNet50 model accuracy and model loss.

DenseNet121 performance in Fig. 6 is comparable to or even better than VGG16. In this case, having dense connections to foster strong gradient flow and recurring feature extraction, DenseNet121 provides efficient and stable training, which is evident from the clarity of its accuracy and loss curves. With outstanding results in important classes like Aircraft Carrier, Cruise, and Submarine, the model always reaches macro and weighted averages at 0.96. By being top-notch in classes with a broad range of data support, such as Recreational and Sailboat, the model demonstrates that it can confidently handle both rich and poor data samples and class imbalances. As can be seen in Fig. 7, ResNet50 is the worst of the four models in general. Its macro and weighted averages of 0.84 signal especially low accuracy in aircraft carrier and bulker type differentiation. It is probable that the training of ResNet50 presents less efficient or less stable convergence, characterized by unstable values of loss and less predictable increases in accuracy. Such behavior can be a result of underfitting or optimization challenges, probably as a result of the inability to exploit the depth of the network when available samples are limited. Although ResNet50 uses residual connections to help in training, it performs relatively poorly in all classes compared to other models in this classification task.

**Fig. 8:**
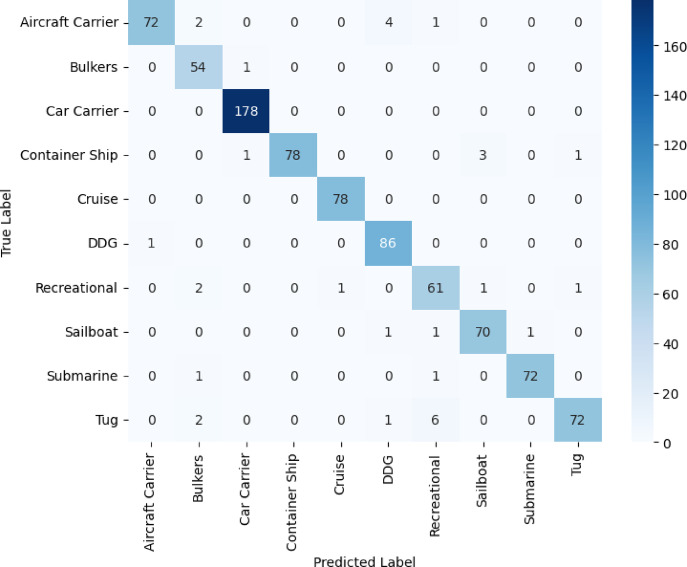
VGG16 confusion matrix.

**Fig. 9:**
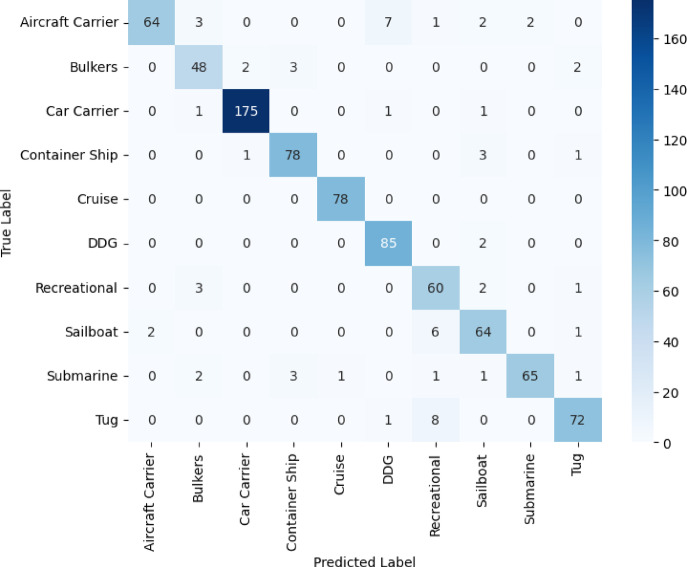
VGG19 confusion matrix.

**Fig. 10:**
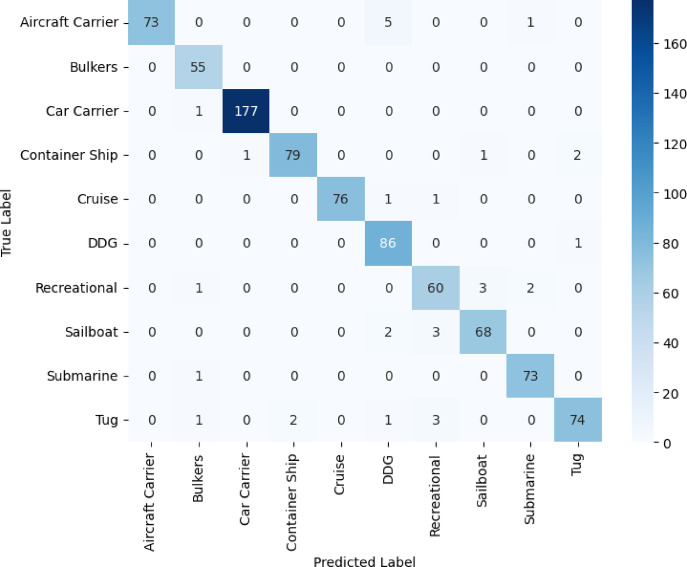
DenseNet121 confusion matrix.

**Fig. 11:**
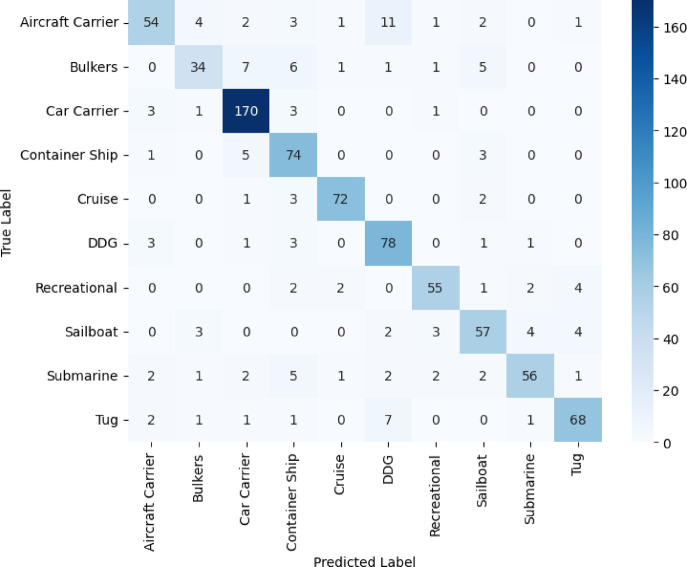
ResNet50 confusion matrix.

In Figs. 8–11, we see the confusion matrices for the four convolutional neural networks, namely VGG16, VGG19, DenseNet121, and ResNet50, and draw fruitful insights about their performance in the classification of vessels. The confusion matrices represent a visual dissection of the models’ ability to distinguish between vessel categories, complementing the table’s precision, recall, and F1-score values. A dominant diagonal in the confusion matrix of the VGG16 shown in Fig. 8 shows that the VGG16 correctly classified most of the vessel images in the right classes. The matrix indicates that misclassifications are uncommon, with VGG16 indicating consistent performance in recognizing both large and small vessel types. This emphasizes the high precision, and F1-scores consistently reported for most vessel categories. Model performance is impressive in that it is capable of generalizing well, and it can accurately discriminate structurally similar vessels such as cruise ships and recreational boats. The results shown in Fig. 9 for VGG19 emphasize more misclassified categories than in the case of VGG16. Although the model does well on some categories, it has clear misclassifications between visually alike groups, such as bulk carriers and container ships or sailboats and recreational craft. As a result, the F1-scores and precision for these classes are to some extent impaired by these misclassifications. The confusion matrix data show that even though a deeper model such as VGG19 is used, the performance is not always reliable for intra-class variations that could be due to overfitting or the model’s greater dependence on subtle visual cues. The confusion matrix of Fig. 10 for DenseNet121 is perfectly aligned, which clearly shows pronounced boundaries when classifying different vessel types. Most of the errors are due to exceptional cases, and the rest of the classifications are correct. The close architecture’s distinctive connection allows the development of more reliable and separable features, which allows the model to provide high recall and F1 scores almost for all vessel categories.

The matrix in Fig. 10 suggests that DenseNet121 is especially good at detecting subtle differences, situated it well as one of the most reliable models for this exercise. In contrast, the confusion matrix in Fig. 11 for ResNet50 has higher errors than those presented by DenseNet121. Dispersion on the matrix of the diagonal indicates confusion about differentiating various classes of boats. For example, big ships can more easily be mistaken for each other than small ones, and the classification of the latter is even more erratic. Such classification mistakes are mirrored in reduced recall and F1 scores during the analysis.

**Fig. 12:**
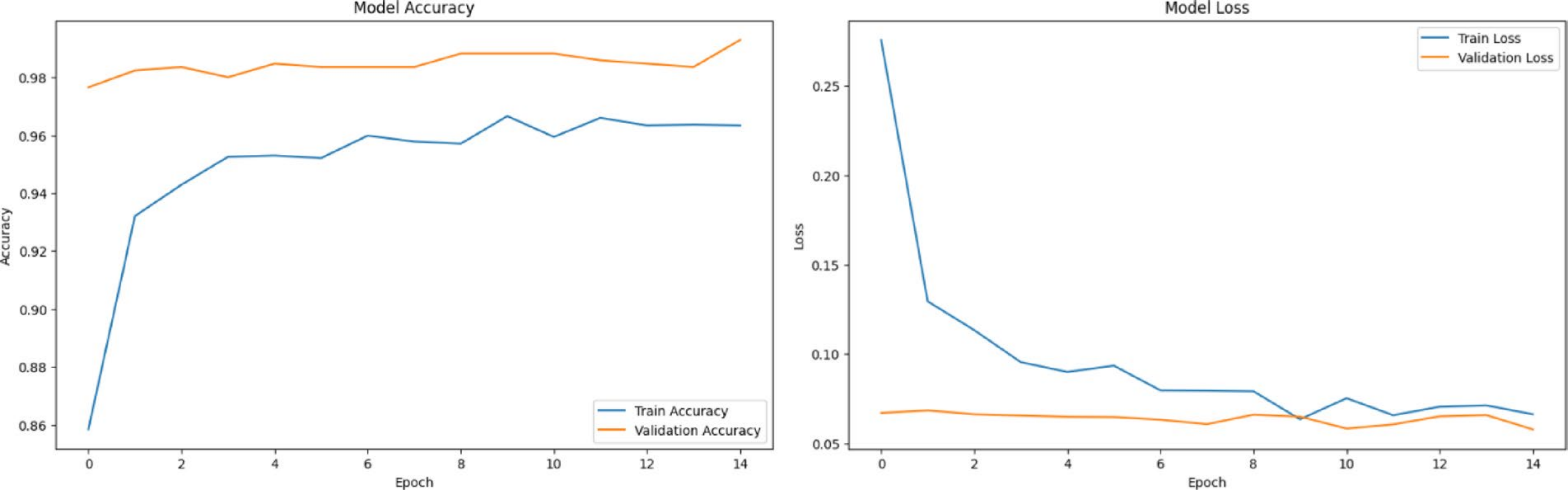
Accuracy and model loss for stacked ensemble learning with a meta-model.

The left subfigure in Fig. 12 shows the training accuracy of the ensemble learning, whereas the right subfigure shows the model loss as a function of the training epochs. The graph shows that during training, the ensemble learning retains consistent accuracy and loss improvements. At the initial phases of training, the model accuracy indicates a quick rise, and the epochs immediately following it experience a gradual and smooth climb, indicating that the model rapidly learns the most important discriminative features and constantly refines their accuracy. The near coincidence of the training and validation accuracy curves provides for the model’s capacity for generalization effectively, indicating low overfitting and reliable accuracy of the model on the data it has not seen before. It is shown on the right, the loss curve declines steadily over epochs for both training and validation sets. It is possible to interpret the sustained decrease in the curve as the model having managed to reduce prediction error during the training period. Repetitive convergence and almost identical training and validation loss suggest the model’s stability and high robustness. Figure 12 shows that the ensemble learning model is very efficient in terms of achieving a good balance between fast training and strong generalization. In contrast to traditional models (e.g., VGG16, VGG19, DenseNet121, and ResNet50), ensemble learning outperforms on stable, reliable performance that minimizes overfitting and training errors that are apparent in other models. The agreement with quantitative estimates, which demonstrate the ensemble learning model’s optimal macro and weighted average precision, recall, and F1-scores, highlights its effectiveness.

**Fig. 13:**
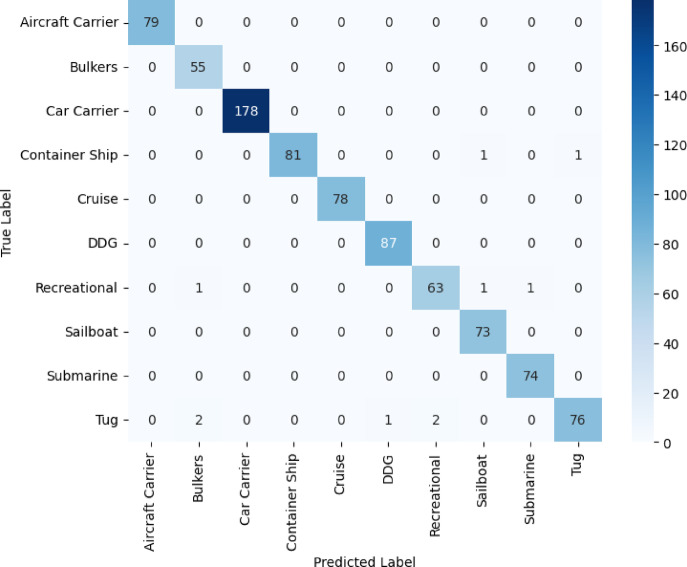
Confusion matrix for stacked ensemble learning with a meta-model.

The confusion matrix used in the ensemble learning model that combines data from several deep learning systems is depicted in Fig. 13. The diagonal dominance of the figure indicates that a large number of vessel images are correctly classified by the model. Small off-diagonal entries show that the ensemble learning framework increases accuracy by aligning the strengths and minimizing the weaknesses of the individual models. Unlike the confusion matrices of the standalone models, the ensemble-learner demonstrates superior discriminative ability in particular class discrimination between classes that previously overlapped. This evidence highlights the advantages of using multiple approaches when addressing complex class distinctions that ultimately increase the reliability and robustness of maritime traffic classification.

**Fig. 14:**
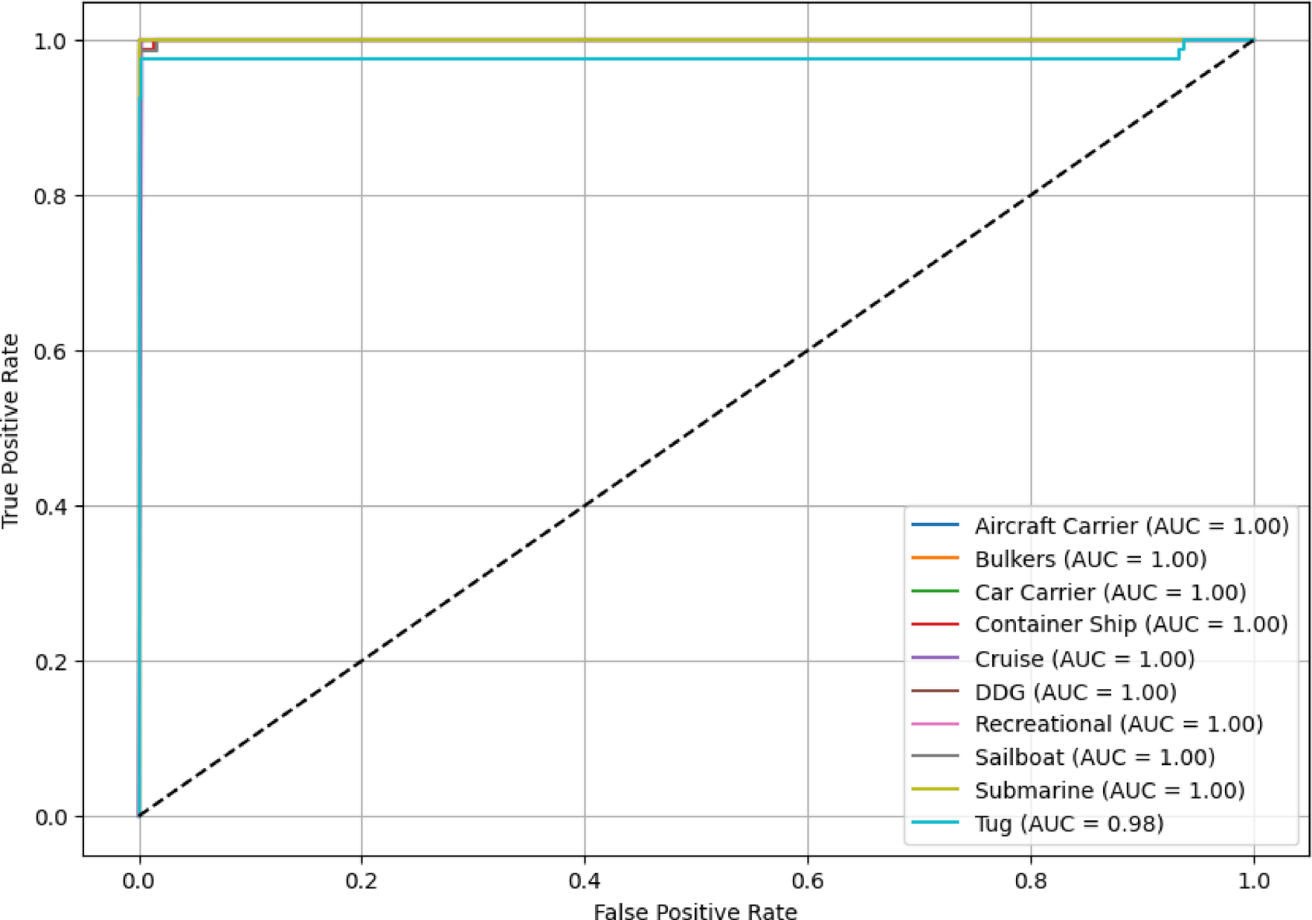
ROC curve for stacked ensemble learning with a meta-model.

Figure 14 is the ROC plot for the ensemble learning model, indicating its ability to distinguish among the ten different vessel types. For most classes (aircraft carrier, bulkers, car carrier, container ship, cruise, DDG, recreational, sailboat, and submarine), the ROC curves run into the upper left quadrant, each with an AUC of 1.0. The model shows outstanding discrimination of these classes, resulting in almost no misclassification or error. Unlike the Tug class, which has an AUC of 0.98 and is said to be very effective, this class does exhibit a slight possibility of mis-categorization of similar vessel types. In all ship classes, the models’ close-to-ideal AUC values confirm that the ensemble learning algorithm is reliable and robust, capable of classifying distinct ship types. This indicates that ensemble-based deep learning exhibits superior capabilities in dealing with multi-class classification issues in the harsh environments of shipping.

**Fig. 15:**
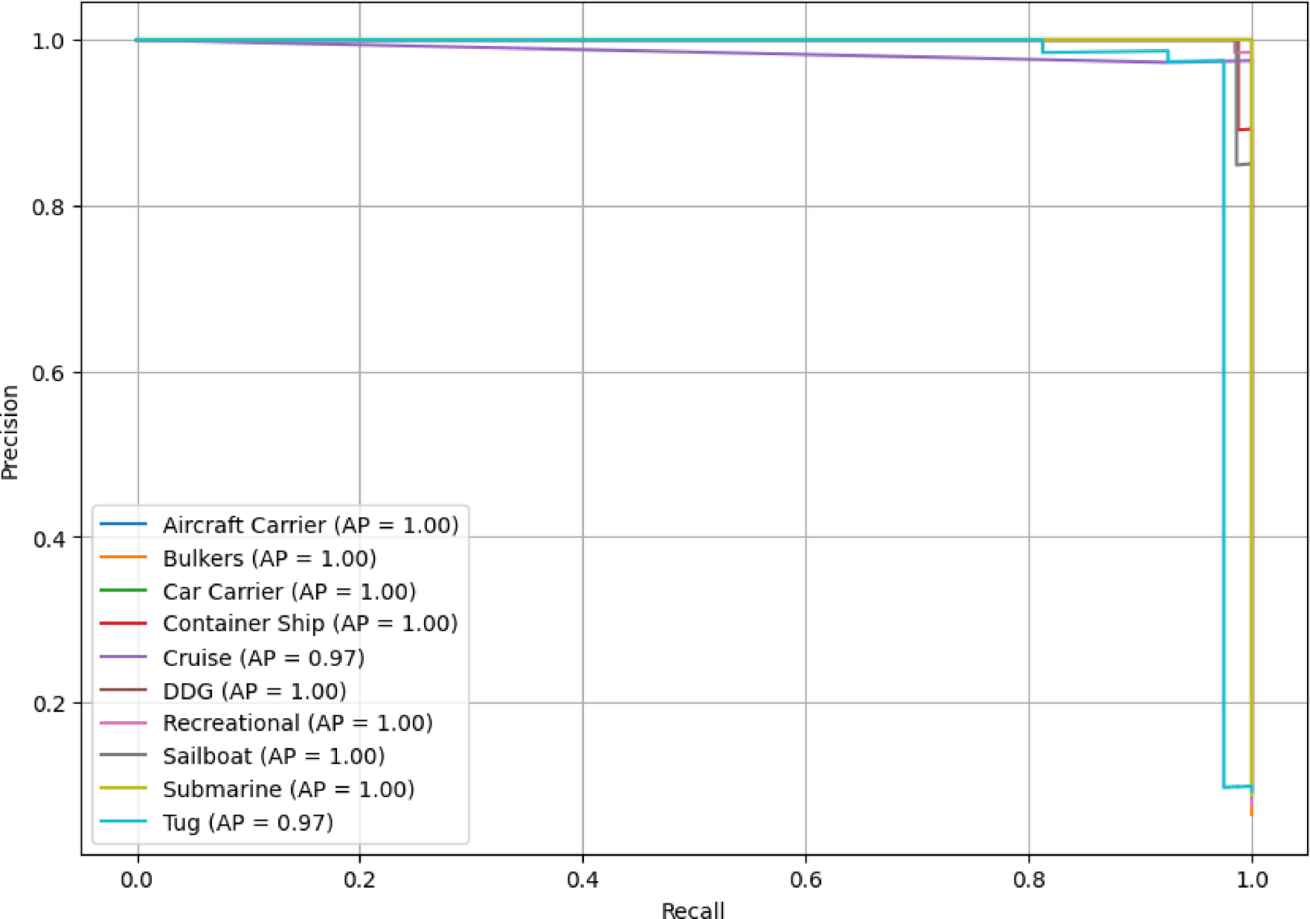
PR Curve for stacked ensemble learning with a meta-model.

The PR curve in Fig. 15 represents the behavior of the ensemble learning model when dealing with imbalanced data in all ten classes of vessels, providing useful information on the capabilities of the model. Each aircraft carrier, bulker, car carrier, container ship, DDG, recreational boat, sailboat, and submarine is represented in the PR curves with AUC values of 1.0, giving perfect precision and recall trade-offs. By attaining an AUC of 1.0 for these classes, the model demonstrates that it is capable of perfectly discriminating true positives and true negatives for all relevant instances. Yet the Cruise and Tug classes have AUC scores of 0.97, which is a small decline in precision or recall compared to other vessel types. Although not perfect, these AUC values exhibit outstanding performance in classifying these vessel types. A portion of this slight decline may be due to the existence of common traits with other classes of vessels or sub-differences between instances of the same category.

**Fig. 16:**
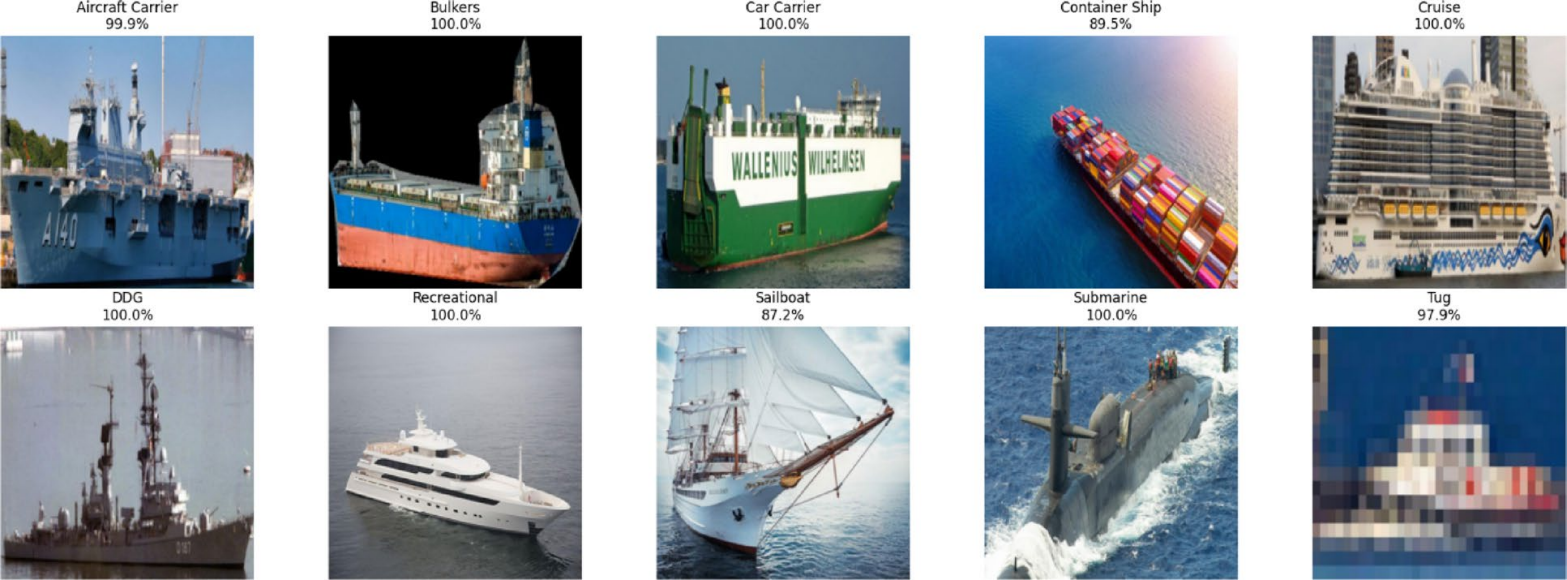
Prediction of sample test images via stacked ensemble learning with a meta-model.

In Fig. 16, it is evident that sample test images are included, and each has been identified by the ensemble learning model with its specified class and associated confidence percentage. Most of the vessel types are assigned very certain classifications by the model, which demonstrates the ability of the model to generalize well to previously unseen instances. For the Bulkers, Car Carrier, Cruise, DDG, Recreational, and Submarine classes, the model predicted 100% confidence, which is the ultimate conviction of the model in determining these vessels. For Aircraft Carrier and Tug, confidence reached 99.9% and 97.2%, respectively, which confirms the model’s persistent and reliable performance. The container ship and sailboat classes, however, had significantly lower confidence rates, i.e., 89.5% and 87.2% confidence, respectively. These reduced confidence scores may reflect the fact that the model was exposed to some visual similarities or characteristic confusions with other vessel types, which resulted in a certain level of confidence uncertainty in the case of these classes.

The research introduces a state-of-the-art architecture that combines ensemble learning with cloud-edge computing, which is intended to improve the precision, efficiency, and immediacy of maritime vessel classification and monitoring. Testing with VGG16, VGG19, DenseNet121, and ResNet50 architectures showed how the ensemble learning approach far outperforms standalone methods in nearly all key metrics. To ensure robustness in dynamic maritime environments, the proposed stacked ensemble learning meta-model is designed to generalize across variations caused by adverse weather, lighting fluctuations, and visual artifacts such as water glare and reflection. During model development, we applied extensive data augmentation techniques that simulate real-world distortions, such as fog, low illumination, haze, rain, and occlusions, ensuring that the learning process captures these variations. By combining multiple deep learning models (VGG16, VGG19, DenseNet121, and ResNet50) and refining their outputs through a meta-model, the framework demonstrates improved adaptability under diverse visual conditions. This structure enables the system to maintain high accuracy and stable performance even when exposed to distorted or degraded maritime imagery.

We used an ensemble of four base models, consisting of two variants (DenseNet and VGG). The predictions from DenseNet121, DenseNet169, VGG19, and VGG16 were combined to make the final decision, which improved both accuracy and robustness. As an ablation study, we tested different ensemble sizes (2, 3, and 4), learning rates, activation functions, and dropout rates to identify and select the configuration that achieved the best performance. The learning rate was set to 1e-4 with the Adam optimizer, ensuring a stable step size for efficient training. The hidden layers employed ReLU activation to introduce non-linearity and enable the model to learn complex patterns, while the output layer used softmax for multi-class ship classification. The ensemble learning model obtained the highest macro average F1-score (0.98), both precision and recall (0.97, 0.98) and excelled over the other models on ROC and PR curves, while achieving an AUC of 1.0 for most classes with only minimal changes in Tug and Cruise classes. Furthermore, the ensemble learning model’s confusion matrix shows that there are few errors, which is a strong indication that the model performs well consistently with vessel types. Furthermore, the sample test images showed high confidence in all class predictions, a few reaching 100% certainty, thereby confirming the suitability of the model for real-world application. This highlights that the proposed framework greatly improves classification accuracy and operational reliability. The role and impact of the key framework components suggest that the study focuses on three core elements: (i) the base deep learning models (VGG16, VGG19, DenseNet121, and ResNet50), (ii) the stacked ensemble learning approach with a meta-model, and (iii) the conceptual integration of cloud-edge hybridization. The results demonstrate that implementing the ensemble learning meta-model, compared to a baseline mechanism, leads to a significant improvement in classification accuracy. This confirms the effectiveness of ensemble learning meta-model in capturing complex feature interactions and enhancing generalization. The cloud-edge hybridization mechanism also provides advantages in terms of reduced latency and improved bandwidth efficiency compared to a purely cloud-based configuration. Its applicability is emphasized in systems requiring prompt vessel identification and classification, i.e., controlling port activities, managing maritime routes, detecting odd traffic patterns, and allowing unmanned ship operation, where low latency, sharp accuracy, and flexibility in adaptation are relevant.

## Conclusion

In this research, a novel stacked ensemble learning with a meta-model-based situation-aware framework with cloud-edge hybridization is developed, aimed at enhancing maritime traffic monitoring and control systems. The framework uses a distributed cloud-edge paradigm to combine the strengths of ensemble learning algorithms, with real-time inference and decision-making occurring at the edge and model training and data management at the cloud. This strategy ensures both fast response times and high model performance under constantly changing maritime conditions. The experimental results demonstrate that the ensemble learning model always outperforms established deep learning methods like VGG16, VGG19, DenseNet121, and ResNet50. For example, the ensemble learning model achieved vessel classification with a macro average F1-score of 0.98 and an accuracy of 0.98, higher than other models that had F1-scores in the range of 0.91–0.96 and accuracies in the range of 0.84–0.96. Specifically, the aircraft carrier, car carrier, submarine, and bulkers were recognized with perfect or close to perfect precision and recall, 1.00 precision, for example, and 0.97 recall. Precision 1.00, Recall 0.97, F1-score 0.99). Moreover, the analysis of the ROC curves showed the high reliability of the ensemble learning model, as 9 out of 10 classes achieved the AUC of 1.00, so the exception was the Tug class with 0.98. In the precision-recall (PR) curve, most classes achieved AUC 1.00, while the cruise and tug classes performed very well, achieving excellent scores of 0.97 even among the weaker results. Sample test predictions illustrate the model’s robust performance with prediction confidences exceeding 99% for most of the ship types, including bulkers, car carriers, DDGs, and submarines, while container ships and sailboats indicate slightly lower confidences at 89.5% and 87.2%, respectively. The proposed framework provides a strong and flexible way to monitor, classify, and control vessels in real-time maritime situations. Due to its reliable and accurate operation on different types of vessels, the proposed framework is suitable for deployment under harsh maritime conditions, e.g., naval security, shipping route logistics, and automation of port systems.

The current study faces certain limitations. The framework does not yet incorporate real-time environmental factors such as weather conditions and maritime traffic density. It may affect its reliability in dynamic operational environments. The present implementation has not been fully optimized for deployment on edge hardware due to which it may create challenges in terms of computational efficiency and scalability. The distributed nature of cloud-edge systems can introduce communication delays that may affect time-critical decision-making, particularly under congested or unstable network conditions. Similarly, edge devices often face hardware constraints such as limited processing power, memory, and energy capacity, which can restrict the scalability and responsiveness of the proposed framework in large-scale maritime environments. Another limitation lies in the limited evaluation of resilience against advanced cyber threats, leaving potential vulnerabilities in adversarial scenarios. In future, this study will be extended to incorporate real-time environmental factors such as weather and the number of maritime traffic to enhance the decision-making abilities. The ensemble learning will also be extended to use reinforcement learning for adaptive route planning and decision-making. We also aim to enhance the compatibility of the framework with real-world edge hardware deployment by optimizing its computational efficiency and scalability, while simultaneously strengthening its resilience against malicious cyber threats.

## Data Availability

The dataset supporting the findings of this study is publicly available on a Kaggle platform with the title, “Ship_classification_dataset” [66] and the link given below: https://www.kaggle.com/datasets/oleksandershevchenko/ship-classification-dataset.
